# Security risk assessment of projects in high-risk areas based on attack-defense game model

**DOI:** 10.1038/s41598-023-40409-w

**Published:** 2023-08-16

**Authors:** Yifan Yao, Wenjing Chen

**Affiliations:** Academy of Information and Network Security, People’s Public Security University of China, Beijing, 100038 China

**Keywords:** Engineering, Mathematics and computing

## Abstract

Assessing the security risk of projects in high-risk areas is particularly important. This paper develops a security risk assessment model for projects in high-risk areas based on the target loss probability model and Bayesian game model. This model is modeled from the perspective of attack-defense confrontation and addresses the issue that traditional risk assessment focuses on the analysis of the attacker yet neglects to analyze the defender—the defender’s optimum defensive information is not quantitatively determined. The risk level, optimum defensive resource value, and optimum defensive strategy of the project are determined through the analysis of a project in the high-risk area. This enables the project’s risk manager to adjust the defensive resources reasonably and optimally, confirming the objectivity and feasibility of the model and offering a new benchmark for security risk assessment, which has significant practical implications.

## Introduction

It is essential to assess the risk of projects in high-risk areas with the economy growing, more and more projects are built and run in high-risk areas. The subsequent hostile threats resulted in significant losses and damages to project employees and assets. But only a limited fraction of security-related studies on projects in high-risk areas are currently available. Such studies, which are comparatively reliable but primarily rely on traditional risk assessment methods^1^ to determine risk levels and safety precaution recommendations, lack quantitative defensive Information about the defender.

The optimum defensive resource value and the optimum defensive strategy for the defender are obtained by the target loss probability model^2,3^ which is based on game theory and the Bayesian game model^4^ from the perspective of attack-defense confrontation, respectively. The target loss probability model, which is based on zero-sum game theory and has a developed application in the field of assessing the risk of terrorist attacks, but the criteria for quantifying and assigning factors in the model need to be improved^5^; the Bayesian game model, which is based on the static game theory of incomplete information, has been developed and widely used in network attack-defense scenarios, but it is rarely used in the field of realistic security risk assessment. In addition, the model’s primary problem is that it is not accurate enough to be used in realistic security risk assessment. The developed model’s relative simplicity, the quantification of attack-defense strategies should be improved, and the model’s priori probability value rely on historical experience, should have a higher objective^6^.

By combining the characteristics of projects in high-risk areas, redefining and quantifying the factors in the target loss probability model, and creating a set of attack-defense strategies under Bayesian game, this paper develops a security risk assessment model for projects in high-risk areas based on the two models and the existing relevant research^7^. In the Bayesian game model, the result of the target loss probability model is utilized as the priori probability value to assess the project's risk in the high-risk area and give objective, quantitative defensive information, including the optimum defensive resources and strategies^8^.

## Methodology

**Figure 1:**
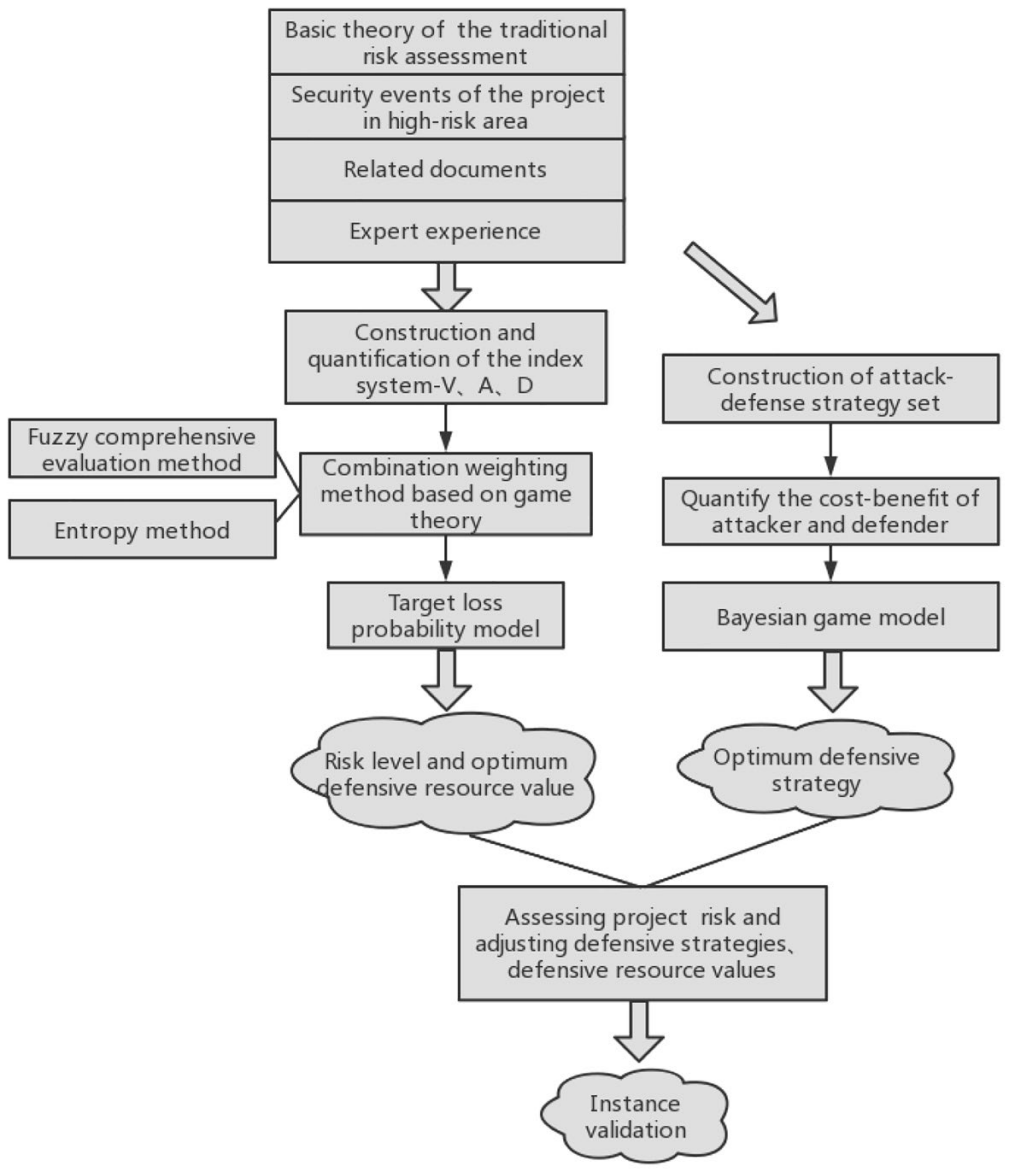
Framework for risk assessment of the projects in high-risk area.

Figure 1 depicts the overall framework of the risk assessment model based on the target loss probability model and the Bayesian game model. To construct and quantify the index system in the target loss probability model—target value V, attacker’s resources A, and defender’s resources D—to ascertain the project risk level and the optimum value for defensive resources, the method uses the cases related to project security in high-risk areas that have been gathered. It also combines traditional risk assessment theory with expert experience. In order to quantitatively determine the optimum defensive strategy for the project, a group of attack—defense strategies for security events in high-risk areas was first constructed and quantified, the research result of the target loss probability model was used as the prior probability value in the Bayesian game model, and then reasonably adjust the defensive resources for the project by the assessed risk level and optimum defensive resource value^9^.

### Construction of target loss probability model

The target loss probability model proposed by Major^2^ is based on the zero sum game—the attack and defense revenue matrix is zero. The problem it discusses and solves is: for a specific target, if terrorists launch an attack with certain resources, and the defender allocates resources to defend the target, how to determine the probability of target loss under the situation of the game between the attacker and the defender. On this basis, this paper applies the target loss probability model to the social security risk assessment of the projects in high-risk areas. The attack and defense parties are the enemies who attack the projects and the security management personnel of the projects. At the same time, combining the characteristics of the attack and defense parties in the social security incidents of the projects, we give new definitions to the factors in this model—target value V, attacker A, defender D, and take social security threat incidents as the target value, The two sides of the attack and defense are hostile elements and security management personnel from the projects. By studying the adversarial game process between the attack and defense sides, the risk level and optimal defense resource value are determined. The improved simplified model is as follows:

- V: The target value, represented by the letter V, is the high-risk area project security event. The loss of consequences brought on by the security event defines the target value V, because it reflects the likelihood and desire of the attackers to launch an attack, and the attractiveness of the security event to the attackers.
- A: The attacker is the enemy side in the security event involving the project in the high-risk area, and the attacker's total resources are A_T_. The attacker chooses the attack target and specific attack resources A to launch the attack, and the attack resources are primarily determined by the level of social security in the host country, the level of social security in the region, the capabilities of terrorist organizations in the region, the capabilities of public order and criminals in the region, ethnic and religious conflicts, and the attractiveness of the project.
- D: The defender is the project's risk manager in the high-risk area, and its total resource allocation is D_T_, which allots defensive resources D to counteract various attacks. The defensive resources are determined based on the safety management organization structure of the project, security personnel strength of the project, security layout and physical protection of the project, technical protection of the project, emergency response, commuter security, sustainable compliance operation capability of the project.
- The risk value is represented by the function ${\text{p(V,A, D)}}$.
- The anticipated loss formula is as follows: The attacker constantly seeks to maximize attack likelihood and consequence loss, while the defender seeks to decrease it.
  1 $${\text{EL}}\;{ = }\;{\text{ V}} \cdot {\text{p(V, A, D)}}$$

Based on this simplified model, it is known that when the defender defends against an assault using specific defensive resources and the attacker uses specific attack resources, the probability of loss for the attacked target is:

2 $${\text{p}}\left( {\text{V,A,D}} \right)\;{ = }\;\frac{{{\text{EL}}}}{{\text{V}}}\;{ = }\;{\text{exp}} - \left( {\frac{{{\text{A}} \cdot {\text{D}}}}{{\sqrt {{\text{V}}_{{}} } }}} \right){{ \times }}\left( {\frac{{{\text{A}}^{{2}} }}{{{\text{A}}^{{2}} {\text{ + V}}}}} \right)$$

$- \frac{{{\text{A}} \cdot {\text{D}}}}{{\sqrt {\text{V}} }}$ denotes the probability of the attacker launching an attack and $\frac{{{\text{A}}^{{2}} }}{{{\text{A}}^{{2}} {\text{ + V}}_{{}} }}$ denotes the probability of the attacker’s successful attack.

When under assault, the defender must continuously allocate defensive resources to the attack target with a high expected loss value (EL) until the attack target’s EL value approaches equilibrium. When the target EL values are equal, at which point $\text{EL } = {\text{ EL}}^{0}$. Assuming that k is a normalization constant and that q_i_ represents the probability that target i will be attacked:

3 $${\text{q}}\;{ = }\;\frac{{ - {\text{k}}}}{{{\text{V}}\frac{\partial }{{\partial {\text{D}}}}{\text{p}}\left( {{\text{V}}_{{}} {\text{,A,D}}} \right)}}\;{ = }\;\frac{{\text{k}}}{{{\text{VA}}^{{0}} {\text{EL}}^{{0}} {\text{V}}^{{ - \frac{{3}}{{2}}}} }}{ = }\frac{{{\text{k}}\sqrt {\text{V}} }}{{{\text{A}}^{{0}} {\text{EL}}^{{0}} }}$$

According to this equation, the attack resources allocated by the attacking side to the target are roughly equal to the open square of the target value. where A^0^ is the optimum attack resource, which can be solved by the following equation:

4 $${\text{A}}^{{0}} {\text{(V,D) = }}\frac{{{\text{V}}^{{\frac{{3}}{{2}}}} }}{{\text{D}}}{ + }\frac{{1}}{{9}}\sqrt {{\text{V}}^{{3}} {{ + 27 }} \times \frac{{\sqrt {3} \cdot {\text{V}}^{{3}} }}{{{\text{D}}^{{2}} }}} - \frac{{1}}{{3}}{{ \times }}\frac{{\text{V}}}{{\frac{{{\text{V}}^{{\frac{{3}}{{2}}}} }}{{\text{D}}}{ + }\frac{{1}}{{9}} \cdot \sqrt {{\text{V}}^{{3}} {{ + 27 }}\times\frac{{\sqrt {3} \cdot {\text{V}}^{{3}} }}{{{\text{D}}^{{2}} }}} }}$$

From Eqs. (2) and (4), the solution equation for the optimum defensive resource is as follows:

5 $${\text{D}}^{{0}} \;{ = }\;\frac{{\sqrt {\text{V}} }}{{{\text{A}}^{{0}} }}\;{{ \times }}\;{\text{ln}}\left[ {\frac{{\text{V}}}{{{\text{EL}}}}{{ \times }}\frac{{\left( {{\text{A}}^{{0}} } \right)^{{2}} }}{{\left( {{\text{A}}^{{0}} } \right)^{{2}} {\text{ + V}}}}} \right]$$

### Construction of Bayesian game model^10^

**Figure 2:**
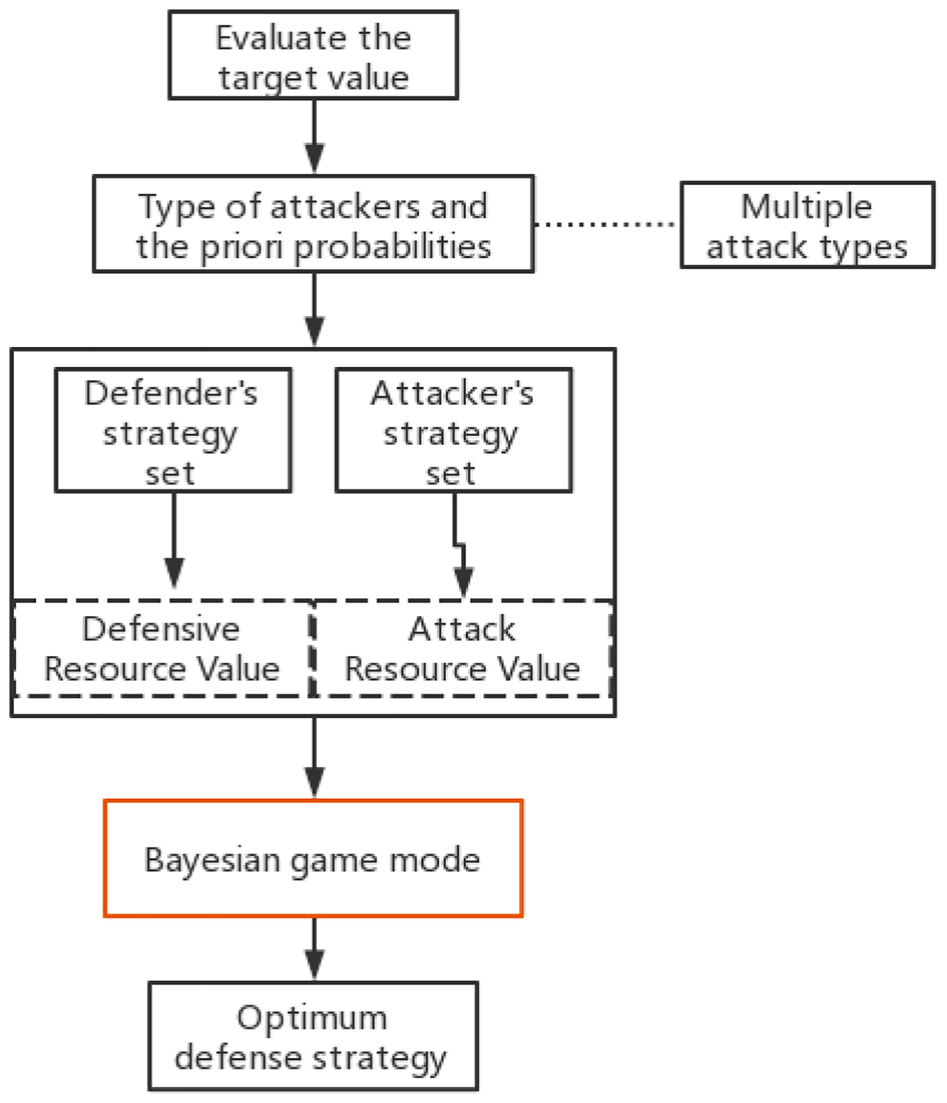
Bayesian game.

Bayesian game model first evaluates the target value, summarizes the types of security events in high-risk areas and behaviors of attackers to construct a strategy set for both attackers and defenders. Then, the modle quantifies the cost- benefit by substituting the attack-defense benefit matrix into the Bayesian framework^11^, at the same time, using the result of the target loss probability model as the priori probability. The Bayesian attack defense game model is shown in Fig. 2.

#### Notations and definitions of Bayesian model model

The relevant symbols and definitions in the Bayesian attack defense game model are shown in Table 1.

**Table 1:** Definition of notation related to Bayesian model.

Variables	Definition	Variables	Definition
A	Attack Strategies	$\mu_{{\text{k}}}$	The prior probability of k types of attackers
D	Defensive Strategies	$U_{{\text{a}}}^{{\text{k}}}$	Expected benefits for k types of attackers
S	Strategy Set	$p_{k}$	Probability of attacker A_k_ attacking the project
$D_{T}$	Total defensive resources	$p_{{k^{*} }}$	Probability of attack on the project under the balanced strategy
$$S_{A}$$$$S_{D}$$	Attack strategy set and Defensive strategy set	$$P_{D}$$	Vulnerability of the project
$A_{k}$	k types of attackers	L	Losses after the project is attacked
$S_{{\text{a}}}^{{\text{k}}}$	A_k_’s strategy set	$G_{{\text{k}}}$	The value of the project in the eyes of attacker A_k_

#### Bayesian strategy set and cost–benefit

The set can be used to represent the attacker's strategy in a high-risk area project attack- defense scenario:

6 $${\text{A}}\;{ = }\;{\text{(a}}_{{1}} {\text{,a}}_{{2}} {,} \ldots {\text{,a}}_{{\text{n}}} {)}$$

7 $$\begin{array}{*{20}c} {s.t.} & {} \\ \end{array} 0 \le a_{1} ,a_{2} , \ldots ,a_{n} \le 1$$

8 $${\text{a}}_{{1}} {\text{ + a}}_{{2}} { + } \cdots + {\text{a}}_{{\text{n}}} = 1$$

9 $${\text{S}}_{{\text{A}}} \;{ = }\;\left. {\left\{ {{\text{A}} \in {\text{R}}^{{\text{n}}} {:}\;{0} \le {\text{a}}_{{\text{i}}} \le {1}\;{\text{ for}}\;{\text{ i}}\;{ = }\;{1,2,} \ldots {\text{,n,}}\mathop \sum \limits_{{\text{i = 1}}}^{{\text{n}}} {\text{a}}_{{\text{i}}} \;{ = }\;{1}} \right.} \right\}$$

This strategy can be described as a model of resource distribution for the defender:

10 $${\text{D}}\;{ = }\;{\text{(d}}_{{1}} {\text{,d}}_{{2}} {,} \ldots {\text{,d}}_{{\text{n}}} {)}$$

11 $$\begin{array}{*{20}c} {{\text{s}}{\text{.t}}{.}} & {{\text{d}}_{{1}} } \\ \end{array} {\text{ + d}}_{{2}} { + } \cdots {\text{ + d}}_{{\text{n}}} \;{ = }\;{\text{D}}_{{\text{T}}}$$

The defender’s strategy set is described as follows:

12 $${\text{S}}_{{\text{D}}} \;{ = }\;\left\{ {\left. {{\text{D}} \in {\text{R}}^{{\text{n}}} {:0} \le {\text{d}}_{{\text{i}}} \le {\text{D}}_{{\text{T}}} \;{\text{for}}\;{\text{ i}}\;{ = }\;{1,2,} \ldots {\text{,n,}}\mathop \sum \limits_{{\text{i = 1}}}^{{\text{n}}} {\text{d}}_{{\text{i}}} \;{ = }\;{\text{D}}_{{\text{T}}} } \right\}} \right.$$

13 $${{\upmu }}_{{\text{k}}} \left( {{\text{0}}\;{\text{ < }}\;{{\upmu }}_{{\text{k}}} \;{{ < }}\;{\text{1,}}\;\;\mathop \sum \limits_{{{\text{k = 1}}}}^{{\text{m}}} {{\upmu }}_{{\text{k}}} \;{{ = }}\;{{1}}} \right)$$

14 $${\text{U}}_{{\text{a}}}^{{\text{k}}} \;{ = }\;\mathop \sum \limits_{{\text{i = 1}}}^{{\text{n}}} {\text{p}}_{{\text{k}}} \times {\text{G}}_{{\text{k}}} \times {\text{P}}_{{\text{D}}} {,}\;{\text{k = 1,2,}} \ldots {\text{,m}}$$

The expected losses and gains of the defender can be expressed as:

15 $${\text{L}}\;{ = }\;\mathop \sum \limits_{{\text{i = 1}}}^{{\text{n}}} \left[ {\mathop \sum \limits_{{\text{k = 1}}}^{{\text{m}}} {\upmu }_{{\text{k}}} {\text{p}}_{{\text{k}}} {\text{LP}}_{{\text{D}}} } \right]$$

16 $${\text{U}}_{{\text{d}}} \,{ = }\; - {\text{L}}$$

By applying the most effective strategy, the attacker maximizes his expected benefit. Attacker A’s optimization problem is expressed as follows:

17 $${\text{maxU}}_{{\text{a}}}^{{\text{k}}} \;{ = }\;{\text{max}}\mathop \sum \limits_{{\text{i = 1}}}^{{\text{n}}} {\text{p}}_{{\text{k}}} \times {\text{ G}}_{{\text{k}}} \times {\text{ P}}_{{\text{D}}} {,}\;\;{\text{k}}\;{ = }\;{1,2,} \ldots {\text{,m}}$$

The defender’s objective is to minimize expected loss by allocating limited defense resources optimally to counter various sorts of attackers.

18 $${\text{maxU}}_{{\text{d}}} \;{ = }\;{\text{minL}}\;{ = }\;{\text{min}}\mathop \sum \limits_{{\text{i = 1}}}^{{\text{n}}} \left[ {\mathop \sum \limits_{{\text{k = 1}}}^{{\text{m}}} {\upmu }_{{\text{k}}} \times {\text{p}}_{{\text{k}}} \times {\text{ L }}\times {\rm P}_{{\text{D}}} } \right]$$

The Nash equilibrium is reached when the attack-defense sides are unable to increase or decrease their benefits by adjusting their strategies. For the complete information game, the Hessani transformation^12^ must be used to solve the static Bayesian Nash equilibrium. As a consequence, the Bayesian Nash equilibrium must satisfy the two following formulas:

19 $$\mathop \sum \limits_{{\text{i = 1}}}^{{\text{n}}} {\text{p}}_{{{\text{k}}^{*} }} {\text{LP}}_{{{\text{D}}^{*} }} \ge \mathop \sum \limits_{{\text{i = 1}}}^{{\text{n}}} {\text{p}}_{{\text{k}}} {\text{LP}}_{{{\text{D}}^{*} }} {\text{,p}}_{{\text{k}}} \in {\text{S}}_{{\text{a}}}^{{\text{k}}} {,}\;{\text{k}}\;{ = }\;{1,2,} \ldots {\text{,m}}$$

20 $$\mathop \sum \limits_{{\text{i = 1}}}^{{\text{n}}} \mathop \sum \limits_{{\text{k = 1}}}^{{\text{m}}} {\upmu }_{{\text{k}}} {\text{p}}_{{{\text{k}}^{*} }} {\text{LP}}_{{{\text{D}}^{*} }} \le \mathop \sum \limits_{{\text{i = 1}}}^{{\text{n}}} \mathop \sum \limits_{{\text{k = 1}}}^{{\text{m}}} {\upmu }_{{\text{k}}} {\text{p}}_{{{\text{k}}^{*} }} {\text{LP}}_{{\text{D}}} {,}\;{\text{d}} \in {\text{S}}_{{\text{d}}}$$

### Combination weighting method based on game theory

The subjective and objective weighting method based on game theory^13^, which combines the respective advantages of the two methods to determine the weights, is founded on the fundamental idea of minimizing the difference between combination weights and individual weights, thereby minimizing the sum of deviations to maximize the interests of both attackers and defenders. The following are the method’s primary solution steps:

•For a basic set of weight vectors $U = \left\{ {u_{1} ,u_{2} , \ldots ,u_{n} } \right\}$, these n vectors are arbitrarily linearly combined into a set of possible weights:

21 $${\text{U}}\;{ = }\;\mathop \sum \limits_{{\text{k = 1}}}^{{\text{n}}} {\upalpha }_{{\text{k}}} {\text{u}}_{{\text{k}}}^{{\text{T}}} \;\;\left( {{\upalpha }_{{\text{k}}} > 0} \right)$$

where u is one possible weight vector of the set of possible weight vectors and $\alpha_{k}$ is a weight coefficient.

•According to game-theoretic principles, the linear combination weight coefficients are optimized by reducing the deviation of u from the individual uk in order to find consensus between the various weights $\alpha_{k}$.

22 $${\text{min}}\left\| {\mathop \sum \limits_{{\text{j = 1}}}^{{\text{n}}} {\upalpha }_{{\text{j}}} \times {\text{ u}}_{{\text{j}}}^{{\text{T}}} - {\text{u}}_{{\text{i}}}^{{\text{T}}} } \right\|_{{2}} \;\;\left( {{\text{i}} = 1,2, \ldots ,{\text{n}}} \right)$$

The first order derivative needed for the optimization of the aforementioned equation can be determined using the matrix differentiation property as follows^14^:

23 $$\mathop \sum \limits_{{\text{j = 1}}}^{{\text{n}}} {\upalpha }_{{\text{j}}} \times {\text{ u}}_{{\text{i}}} \times {\text{ u}}_{{\text{j}}}^{{\text{T}}} \;{ = }\;{\text{u}}_{{\text{i}}} \; \times {\text{ u}}_{{\text{i}}}^{{\text{T}}} \;\;\left( {{\text{i}} = 1,2, \ldots ,{\text{n}}} \right)$$

which corresponds to the system of linear equations:

24 $$\left[ {\begin{array}{*{20}c} {{\text{u}}_{{1}} \cdot {\text{u}}_{{1}}^{{\text{T}}} } & {{\text{u}}_{{1}} \cdot {\text{u}}_{{2}}^{{\text{T}}} } & \cdots & {{\text{u}}_{{1}} \cdot {\text{u}}_{{\text{n}}}^{{\text{T}}} } \\ {{\text{u}}_{{2}} \cdot {\text{u}}_{{1}}^{{\text{T}}} } & {{\text{u}}_{{2}} \cdot {\text{u}}_{{2}}^{{\text{T}}} } & \cdots & {{\text{u}}_{{2}} \cdot {\text{u}}_{{\text{n}}}^{{\text{T}}} } \\ \vdots & \vdots & \vdots & \vdots \\ {{\text{u}}_{{\text{n}}} \cdot {\text{u}}_{{1}}^{{\text{T}}} } & {{\text{u}}_{{\text{n}}} \cdot {\text{u}}_{{2}}^{{\text{T}}} } & \cdots & {{\text{u}}_{{\text{n}}} \cdot {\text{u}}_{{\text{n}}}^{{\text{T}}} } \\ \end{array} } \right]\left[ {\begin{array}{*{20}c} {{\upalpha }_{{1}} } \\ {{\upalpha }_{{2}} } \\ \vdots \\ {{\upalpha }_{{\text{n}}} } \\ \end{array} } \right]{ = }\left[ {\begin{array}{*{20}c} {{\text{u}}_{{1}} \cdot {\text{u}}_{{1}}^{{\text{T}}} } \\ {{\text{u}}_{{2}} \cdot {\text{u}}_{{2}}^{{\text{T}}} } \\ \vdots \\ {{\text{u}}_{{\text{n}}} \cdot {\text{u}}_{{\text{n}}}^{{\text{T}}} } \\ \end{array} } \right]$$

• After deriving $\left( {{\upalpha }_{{1}} {,}\;{\upalpha }_{{2}} {,}\; \ldots {,}\;{\upalpha }_{{\text{n}}} } \right)$ from this equation, it is normalized to:

25 $${\upalpha }_{{\text{k}}}^{*} { = }\frac{{{\upalpha }_{{\text{k}}} }}{{\mathop \sum \nolimits_{{\text{k = 1}}}^{{\text{n}}} {\upalpha }_{{\text{k}}} }}$$

The final weights are derived as follows:

26 $${\text{u}}^{*} { = }\mathop \sum \limits_{{\text{k = 1}}}^{{\text{n}}} {\upalpha }_{{\text{k}}}^{*} \cdot {\text{u}}_{{\text{k}}}^{{\text{T}}}$$

### Construction of index system and attack-defense strategy set for projects in high-risk area

#### Construction of V, A, D index system

The methods used to construct the index system of each factor in the target loss probability model are literature collection and case study, and the construction principles should follow objectivity, typicality, operability and hierarchy. To this end, it should not only refer to the general attack-defense resource index system—the fundamental theory of risk assessment, but also combine the characteristics of projects in high-risk areas with real case studies. Therefore, when constructing the index system, it should also take into account the characteristics of projects in high-risk areas, realistic cases, pertinent literature, pertinent official websites, and the experience and knowledge of experts for revision in addition to referencing the general attack-defense resource index system-the basic theory of risk assessment. The target loss probability model is used to determine the risk level and the optimum defensive resource value on the basis of this index system, and the values of V, A, and D are determined by a combination weighting method based on game theory (fuzzy comprehensive evaluation method^15^ and entropy value method^16^). The indicators are constructed as depicted in Table 2.

**Table 2:** V, A, D indicators of projects in high-risk area.

Primary indicators	Secondary indicators	Tertiary indicators
Indicators of the attacker-A	Level of social security in the host country	Social security level
		Life safety level
		Economic security level
		Production security level
	Level of social security in the region	Terrorist attack
		Mass unexpected incident
		Law and order crime
		War and armed conflict
		Kidnapping for ransom
	Capability of terrorist organizations in the region	Number of enemies
		Key persons involved in terrorism
		Terrorist organization ideology
		Social background and intent
		Actions and capabilities
		Weapons
		Historical terrorist event activity in the region
	Capability of public order and criminal in the region	Number of enemies
		Enemy type
		Enemy purpose
		Target of attack
		Attack strategy
		Weapons
		Activity of historical public order and criminal offense in the region
	Ethnic and religious conflicts	Ethno-religious type
		Ethno-religious conflict level
		Activity of ethnic and religious conflicts
	Attractiveness of the project	Project popularity
		Number of employees
		Project scale and importance
		Important assets and dangerous goods storage level
		Historical attack interest against the project
Indicators of the defender-D	Safety management organization structure of the project	Security management organization structure
		Security management related system
	Security personnel strength of the project	Number of project security personnel
		Competence and quality
		Education and training
		Security personnel qualification level
	Security layout and physical protection of the project	Project building layout
		Project building specific protection elements
		Project perimeter barrier
		Project internal and external protection distance
		Important assets protection in the project
	Technical protection of the project	Intrusion alarm system
		Video surveillance system
		Entrance and exit control system
		Explosion-proof security check system
	Emergency response	Emergency plan
		Emergency communication
		Emergency materials
		Emergency drill
		Emergency shelter
	Commuting security	Commuting security management
		Commuting safety situation
		Relations with local communities
		Local public security departments
		Medical and fire departments
		Other related items
		Emergency rescue traffic
	Sustainable compliance operation capability of the project	Compliance with relevant laws and regulations
		Respect for local culture and customs
		Relationship with local communities
		Transparency in project governance
		Assume operational social responsibility
Indicators of the target-V	Degree of injury to target personnel	
	Degree of exposure of target personnel	
	Degree of damage to target assets	
	Degree of damage to target project operations	
	Target project reputation impact range	
	Scope of environmental impact of the target project	

The following table provides instances of indicator quantification, with the quantification of indicators for both attackers and defenders set at three levels and the quantification of indicators for target values set at five levels. Table 3 describes the quantitative grading of social security indicators, while Table 4 describes the quantitative grading of indicators for the degree of functional operation damage of the target project^17^.

**Table 3:** Index level description of social security level.

Social security level	Index level
The proportion of major and malignant criminal cases in the host country is less than 10%	Low
The proportion of major and malignant criminal cases in the host country ranges from 10 to 30%	Medium
More than 30% of the country’s major and malignant criminal cases	High

**Table 4:** Index level description of degree of damage to project operations.

Degree of damage to target project operations	Index level
Function operation is not affected	None
Some functions operate normally, simple adjustments can return to normal	Slight
Function operation is stopped, small-scale adjustment can be restored to normal	Moderate
Function operation is stopped, large-scale adjustment can be restored to normal	Heavy
Function operation is stopped and cannot be restored	Severe

#### Construction of Bayesian game strategy set and quantification of the cost–benefit

The construction of the attack and defense strategy set in the Bayesian attack and defense game model and the quantification of cost–benefit grading are based on the DMAT classification method in the field of network attack and defense. The attack-defense resource index system is the major reference point for constructing the Bayesian attack-defense game strategy set for the project. In addition, it is also associated with realistic attack-defense cases. The attack-defense cost–benefit values have a range of 0–100, and the type of attackers is subdivided into public order/criminal criminals^18^ and local armed elements/terrorists^19^ based on the severity of the injury. The attack strategy is developed from the standpoint of typical strategies used in realistic cases, the cost of attack is taken into account from the enemy's weaponry, strategy, and type, and the benefit to both the attackers and defender is taken into account from the severity of the damage the enemy has caused. Split the cost–benefit of public order/criminal criminals into five categories, and local armed elements/terrorists into four categories. Table 5 provides the quantified cost benefit values for the attacking party.

**Table 5:** Quantification of the cost–benefit of the attacker’s strategy.

Type of attacker	Classification of attack costs and consequences	Costs	Benefits	Examples of attack strategy
Law and order/criminal elements	The enemy’s goal is straightforward, it uses few, simple resources without careful planning, and it has minimal effects on the project	0–20	0–30	Demonstration riots/Protests
	The enemy's goal is financial gain, it use a small number of simple techniques with well-planning, and it has some effects on the project	21–40	31–50	Kidnapping for extortion
	The enemy's goal is financial gain, it use a select few specialist tools with well-planning, and endanger the assets of the project	41–50	31–50	Theft
	The enemy's goal is financial gain or social connections, it use a select few specialist weapons and tools with well-planning, and results in casualties among the staff of the project	51–70	51–70	Assassination/Shooting
	The enemy's goal is to influence society by well-planned use of explosives, bombs, and other powerful fatal weapons that will have a negative impact on the project	70–100	71–100	Explosion
Local armed elements/terrorists	The enemy’s goal is financial gain or to influence society, it use weapons and equipment with well-planning, results in casualties among the staff of the project and more destructive power than criminal and public security crimes	0–50	0–60	Kidnapping for ransom
	The enemy is for ethnoreligious or political ends, it use firearms and other weapons with well-planning, results in casualties among the staff of the project and more destructive power than criminal and public security crimes	51–60	61–70	Assassination/Shooting
	The enemy is for ethnoreligious or political ends or to exert social influence, it use weapons, bombs, and explosives with well-planning, results in casualties among the staff and property losses of the project and harm to project operations	61–80	71–100	Explosion/Bombing
	The enemy is for ethnoreligious or political ends or to exert social influence, it use weapons, guns,bombs, and explosives with well-planning, results in casualties, the loss of property and facilities, and harm to the project’s ability to function	81–100	71–100	Armed Attack

Set the cost of each benchmark defensive strategy to range from 0 to 10, and the overall cost of defense to be 100. Based on the attack strategies, considering the difficulty and consequences of defending the attack behavior, and taking the project physical protection, technical protection, and security personnel protection strategies as the benchmark, an attack strategy oriented defense strategy is proposed to determine the cost–benefit of attack—defense. Table 6 describes the defense strategy set and costs.

**Table 6:** Defensive strategy set.

Defense category	Defense strategy	Strategy description	Defense cost
Project physical protection	Building layout	Important assets and densely populated areas of the project are located in the core of the building, away from the edges of the building and other weak protection locations	0–10
	Perimeter physical barrier	The main functions of physical perimeter barriers are warning, deterrence, blocking, delay, retardation, preparedness and protection, such as the anti-damage, anti-climbing and permeability of physical fences	0–10
	Set up protection distance	The protective belt is set outside the building of the project to play the role of delay, prevention and protection	0–10
	Building specific protection elements	Reasonable setting of specific building elements, such as bullet-proof glass, anti-theft windows, explosion-proof partitions, hidden observation towers, anti-climbing facilities at the top of fences, and wall reinforcement at the core facilities of the project	0–10
Project technical protection	Intrusion alarm system	Reasonable and strict installation of intrusion alarm systems at key entrances and exits or fences of the project to detect and respond to enemy intrusion in a timely manner	0–10
	Video surveillance system	Reasonable and strict setting of intelligent video monitoring system, timely detection and response to intrusion, and record storage	0–10
	Entrance and exit control system	Reasonably set up entrance and exit control systems, mainly for personnel and vehicle identity authentication, to prevent the entry of suspicious personnel and vehicles or forced entry	0–10
	Explosion-proof security check system	Explosion-proof barriers are set up outside the public areas of the building to detect dangerous goods being transported in and out of the area, and fire extinguishing devices are also provided	0–10
Protection of project security personnel	Security personnel quality	Regular training and drills for security personnel to ensure the quality of security personnel	0–10
	Emergency response and disposal	Emergency response to emergency threats, use of emergency communications for external security forces, and post-event improvement work	0–10

The Bayesian attack-defense game can be solved using the attack-defense benefit matrix and the prior probability value after determining the cost–benefit ratios of attack and defense. The attack-defense benefit matrix and the prior probability value are input into the gambling game software^20^ to solve the Bayesian Nash equilibrium-optimum defensive strategy.

## Results and discussion

### Case validation

This study chooses a project in a high-risk location and conducts a case validation through field trips, conferences, and data collection, in order to confirm the validity of the model and the logic of the risk assessment procedure.

**Figure 3:**
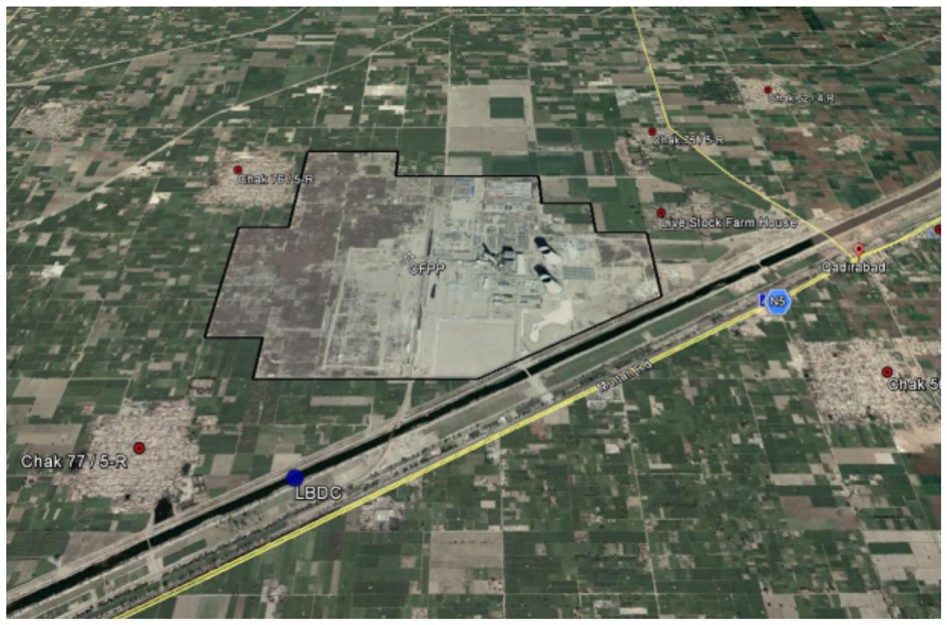
Aerial view of project layout.

The project's basic setup is as follows: it encompasses an area of approximately 6000 acres, has a perimeter of approximately 10 km, 30 fence posts in total, three entrances and exits, and a regular, roomy surrounding road that is close to the local public safety department, fire department, and medical department. SPU (police) is responsible for guarding the project perimeter and protecting employees during their intermediary transportation, while security is responsible for the internal security work of the project, mainly maintaining order and dealing with emergencies. The project is placed in an extremely dangerous and complex social security environment, and its personnel precautions, physical precautions, technical precautions, and emergency response and disposal measures are either inadequate or generic. The project layout is shown in Fig. 3.

The project’s risk assessment is validated based on the built security risk assessment model of the project in the high-risk area and the real scenario of the project, with the results as follows.

### Results and discussion

#### Results of target loss probability model of the project

##### Combination weighting based on game theory of V, A, D

Table 7 shows the result values of the attacker’s resource value A.

**Table 7:** The value of attacker’s resource—A.

Attack indicators	Fuzzy comprehensive evaluation method	Entropy value method	Combined weighting method	Score (rank)
Social security level	0.023	0.0299	0.024952635	3
Life safety level	0.024	0.0299	0.025669644	2
Economic security level	0.022	0.0346	0.02556568	3
Production security level	0.022	0.0346	0.02556568	3
Terrorist attack	0.021	0.0429	0.027197492	3
Mass unexpected incident	0.025	0.0346	0.027716709	2
Law and order crime	0.021	0.0429	0.027197492	3
War and armed conflict	0.022	0.0346	0.02556568	3
Kidnapping for ransom	0.024	0.0299	0.025669644	2
Number of enemies	0.057	0.0118	0.044208829	2
Key persons involved in terrorism	0.057	0.0118	0.044208829	2
Terrorist organization ideology	0.022	0.0346	0.02556568	3
Social background and intent	0.038	0.0236	0.033924937	3
Actions and capabilities	0.022	0.0346	0.02556568	3
Weapons	0.023	0.0299	0.024952635	3
Historical terrorist event activity in the region	0.021	0.0429	0.027197492	3
Number of enemies	0.042	0.0365	0.040443552	2
Enemy type	0.038	0.0236	0.033924937	3
Enemy purpose	0.038	0.0159	0.03174591	3
Target of attack	0.022	0.0346	0.02556568	3
Attack strategy	0.038	0.0236	0.033924937	3
Weapons	0.021	0.0429	0.027197492	3
Activity of historical public order and criminal offense in the region	0.038	0.0236	0.033924937	3
Ethno-religious type	0.023	0.0299	0.024952635	3
Ethno-religious conflict level	0.021	0.0429	0.027197492	3
Activity of ethnic and religious conflicts	0.021	0.0429	0.027197492	3
Project popularity	0.026	0.0429	0.03078254	2
Number of employees	0.122	0.0299	0.095936574	1
Project scale and importance	0.023	0.0299	0.024952635	3
Important assets and dangerous goods storage level	0.025	0.0346	0.027716709	2
Historical attack interest against the project	0.059	0.0429	0.054443853	2
Overall Score	2.489162882			

Table 8 shows the result values of the defender’s resource value D.

**Table 8:** The value of defender’s resource—D.

Defense indicators	Fuzzy comprehensive evaluation method	Entropy value method	Combined weighting method	Score (rank)
Security management organization structure	0.036	0.0336	0.034402731	3
Security management related system	0.039	0.0416	0.040730375	3
Number of project security personnel	0.027	0.0336	0.03139249	2
Competence and quality	0.024	0.0416	0.035713307	2
Education and training	0.029	0.0290	0.029	2
Security personnel qualification level	0.036	0.0336	0.034402731	2
Project building layout	0.027	0.0336	0.03139249	2
Project building specific protection elements	0.03	0.0114	0.017621165	2
Project perimeter barrier	0.027	0.0229	0.024271332	2
Project internal and external protection distance	0.025	0.0416	0.036047778	2
Important assets protection in the project	0.027	0.0229	0.024271332	2
Intrusion alarm system	0.041	0.0416	0.041399317	1
Video surveillance system	0.039	0.0229	0.028284987	3
Entrance and exit control system	0.041	0.0229	0.028953929	1
Explosion-proof security check system	0.041	0.0416	0.041399317	1
Emergency plan	0.036	0.0336	0.034402731	3
Emergency communication	0.025	0.0416	0.036047778	2
Emergency materials	0.036	0.0290	0.031341298	1
Emergency drill	0.041	0.0229	0.028953929	1
Emergency shelter	0.041	0.0229	0.028953929	1
Commuting security management	0.027	0.0229	0.024271332	2
Commuting safety situation	0.029	0.0290	0.029	2
Relations with local communities	0.029	0.0155	0.020015361	2
Local public security departments	0.042	0.0229	0.0292884	2
Medical and fire departments	0.03	0.0114	0.017621165	2
Other related items	0.03	0.0114	0.017621165	2
Emergency rescue traffic	0.025	0.0416	0.036047778	1
Compliance with relevant laws and regulations	0.025	0.0416	0.036047778	2
Respect for local culture and customs	0.025	0.0416	0.036047778	2
Relationship with local communities	0.025	0.0416	0.036047778	2
Transparency in project governance	0.025	0.0416	0.036047778	2
Assume operational social responsibility	0.022	0.0543	0.04349658	2
Overall Score	1.901843005			

Table 9 shows the result values of the value of target’s resource V.

**Table 9:** The value of target’s resource—V.

Target indicators	Fuzzy comprehensive evaluation method	Entropy value method	Combined weighting method	Score (rank)
Degree of injury to target personnel	0.232	0.0935	0.166406884	3
Degree of exposure of target personnel	0.12	0.2123	0.163712957	3
Degree of damage to target assets	0.124	0.1939	0.157104395	4
Degree of damage to target project operations	0.136	0.2449	0.187574659	4
Target project reputation impact range	0.121	0.1202	0.120621123	3
Scope of environmental impact of the target project	0.267	0.1352	0.204579981	2
Total score	3.14009907			

##### Results of target loss probability model

**Figure 4:**
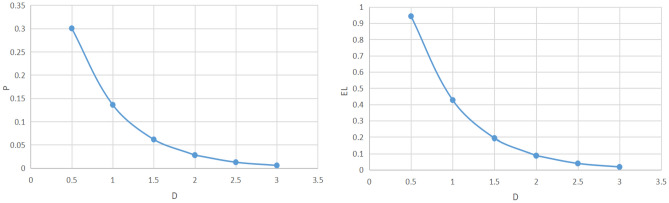
Probability value P and loss expectation EL with different defensive resources.

In order to do dynamic analysis, the target value—V, attacker’s resource—A, and defender’s resource—D are input into the target loss probability model. The results are shown in Table 10 and Fig. 4.

**Table 10:** Results of target loss probability model of the project.

V	A	D	P(V,D,A)	D^0^	EL
3.14009907	2.489162882	1.901843005	0.032543492	0.184699128	0.102189788

The adjustment of defense resource values will cause the probability of target loss to change, thereby affecting the risk level faced by the project. Therefore, the risk level can be divided based on the defense resource values. Figure 4’s target loss probability model results show that when defensive resources increase from 0.5 to 1.5, the target loss probability value is high and the project risk level is at its highest; by increasing defensive resources, the target loss probability value can be effectively reduced, lowering the risk level; when defensive resources from 1.5 to 2, the target loss probability value significantly decreases, and at this time, the project risk level is at its medium; when defensive resources are increased from 2 to 3, the target loss probability value and project risk level are the lowest,but there is no discernible change in the target loss probability value with the increase of defense resources, and the defense resources are now excessive. This is further supported by the expected loss EL result graph, which shows that the expected loss value reduces noticeably when defensive resource levels rise from 0.5 to 2, but does not alter much as defensive resource levels rise further. Combined with the target loss probability result value in Table 10, it can be seen that the risk level of the project is medium, and the optimum defensive resource should be increased by D^0^ over the basic defensive resource. Based on the results of the risk assessment, while considering the weaknesses in the actual safety prevention of the project, the optimum defense resource A^0^ is increased for the three aspects of the video surveillance system, the outlet control system and the quality of the security personnel, thereby reducing the target loss probability value. At this time, the target loss probability value is 0.024282469, with a low risk level, and there is no problem of excessive defense resources.

The existing literature on project risk assessment in high-risk areas is mostly based on traditional risk assessment theories to assess risk levels, such as Varbuchta et al.^21^, which expands the risk variables in large-scale transportation infrastructure projects on the basis of traditional risk assessment and applies them to the executed project assessment process; Li et al.^22^ combined risk management and public safety triangle theory to assess the risk level from vulnerability, threat, and key factors. In this paper, the target loss probability model is applied to the traditional risk assessment process, and indicators are constructed from the perspective of an attack-defense game in combination with realistic attack-defense scenarios. While obtaining the risk assessment level, the optimum defensive resource value facing the attacker is given.

#### Bayesian game model results of project

##### Quantitative values of the cost–benefit of attack and defense

Table 11 shows the quantification of attack and defense costs and benefits.

**Table 11:** Quantification of the cost–benefit of the attack-defense strategies.

Attacker type	Attack strategy	Attack costs	Defend strategies	Defense costs	Attack—defense benefits
Law and order/criminal elements	S_a1_ Demonstration riot/Protests	15	S_d1_	20	20
	S_a2_ Kidnapping for extortion	40	S_d2_	40	50
	S_a3_ Theft	45	S_d3_	40	50
	S_a4_ Assassination / Shooting	55	S_d4_	60	70
	S_a5_ Explosion	70	S_d5_	70	80
Local armed elements/terrorists	S_a6_ Kidnapping for ransom	50	S_d6_	40	60
	S_a7_ Assassination/Shooting	60	S_d7_	60	70
	S_a8_ Explosion/Bombing	75	S_d8_	70	90
	S_a9_ Armed attack	85	S_d9_	80	90

##### Mixed strategy Nash equilibrium solution—gambit software analysis

**Figure 5:**
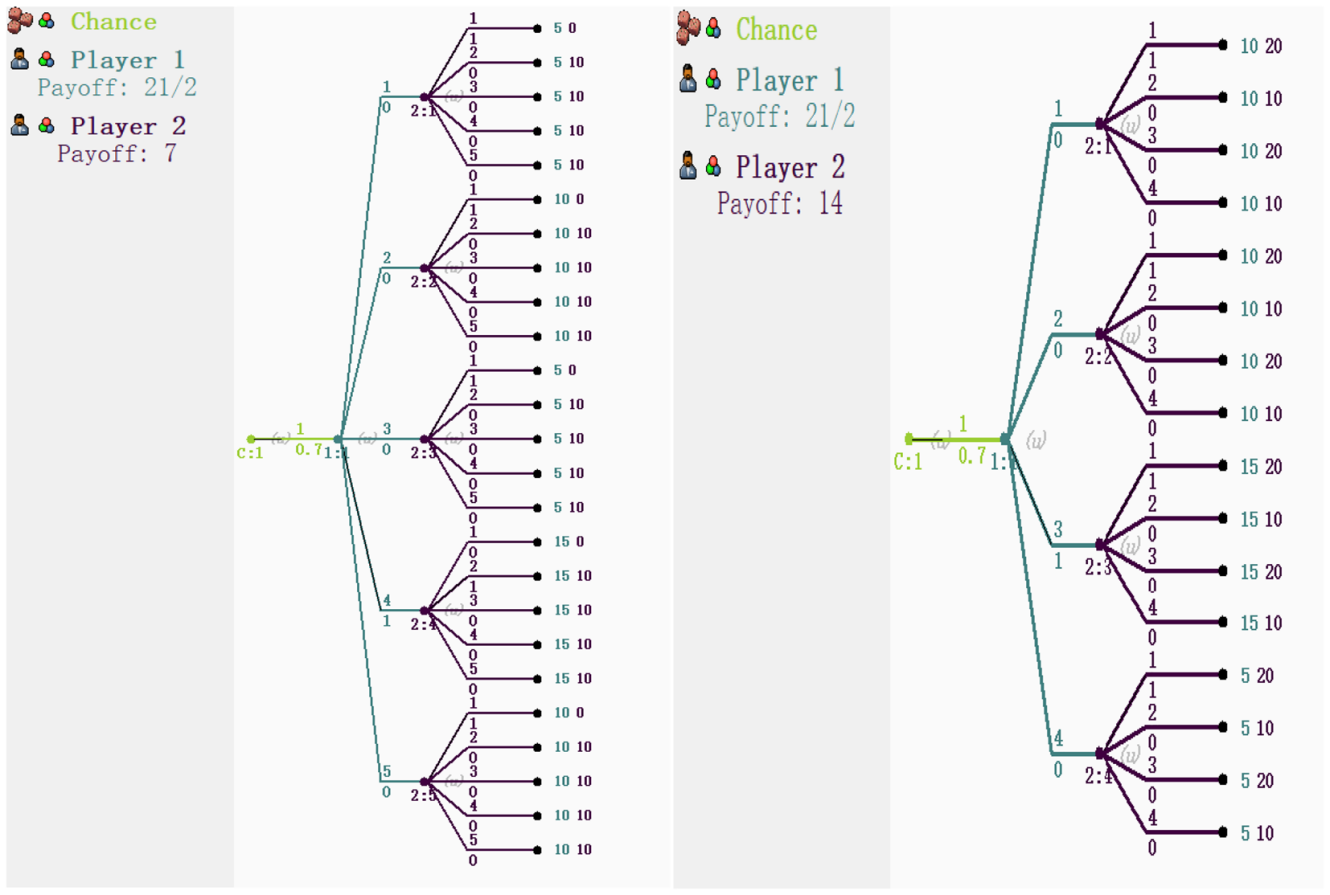
Game tree of attack-defense under different types of attacks.

**Figure 6:**
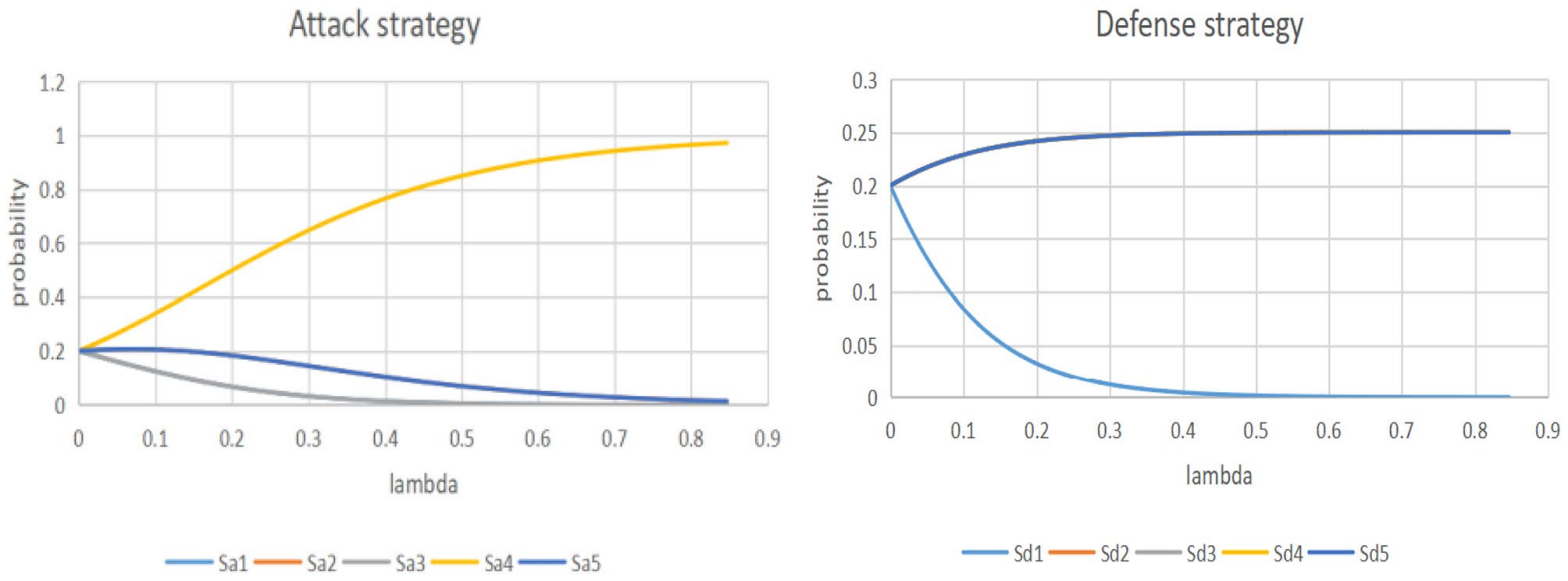
Nash equilibrium of the game under the type of law and order/criminal elements.

**Figure 7:**
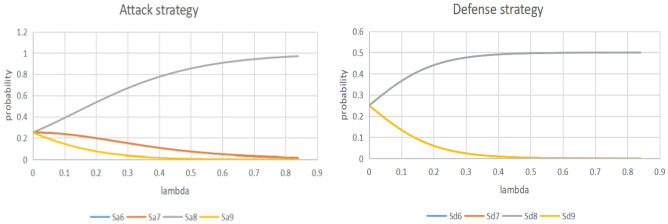
Nash equilibrium of the game under the type of local armed elements/terrorists.

Figure 5 shows the attack and defense game tree constructed under different attacks. Figures 6 and 7 show the Nash equilibrium solutions for different types of attackers, respectively.

The Bayesian attack-defense game model’s findings show that S_a4_ assassination/shooting, S_d2_, is the most likely attack-defense strategy when confronting law and order/criminal elements, while S_a8_ explosion/bombing, S_d6_, is the most likely attack-defense strategy when confronting local armed elements/terrorists. At this point, the attack-defense games have each reached mixed strategy Nash equilibrium. The project security manager should increase D^0^ for the resources in strategies S_d2_ and S_d6_, respectively, in conjunction with the target loss probability model's optimum defensive resource value. This will not only significantly lower the project risk level, but will also maximize the sensible allocation of the optimum defensive strategy and resource value.

The prior probability value in existing Bayesian game models primarily relies on expert experience and historical data, and is mostly applied to network attack-defense game scenarios. For instance, Liu et al.^23,24^ proposed a generalized method of perfect Bayesian Nash equilibrium (BNE) for solving actual network attack-defense; Wang et al.^25^ combined Bayesian game theory and Markov decision methods to construct an incomplete information stochastic game model. However, the model in this study uses the target loss probability result as the attacker's prior probability value, combining the traits of high-risk projects to create Bayesian attack-defense games with various attacker types in realistic attack-defense scenarios, and more objectively determining the optimum defensive strategy against various attackers.

## Conclusion

This paper constructs a security risk assessment model for projects in high-risk areas from the perspective of an attack-defense confrontation game to determine the risk level and optimum defensive information, and verifies the scientificity of the model using the example of a project in a high-risk area, which is based on the traditional risk assessment theory, the target loss probability model, and Bayesian game model. This model combines the characteristics of both attack and defense sides in project security in high-risk areas, innovatively taking security events and resources of both attack and defense sides as factors in the target loss probability model, and quantitatively assigning values based on these factors to obtain relevant risk values; at the same time, the types and strategies of attackers are classified to construct a Bayesian game model, and the target loss probability result is taken as its prior probability to obtain the optimum defensive strategy.

The model is modeled from the perspective of offensive defense confrontation game, solves the problem of focusing on the attacker’s analysis in the traditional risk assessment and defending’s insufficient analysis, quantitatively determines the defense’s optimum defense information, while solving the issue of unclear defense strategy classification in the previous Bayes offensive game model in the target loss probability model, the precursor probability value is not quantitative, the lack of consideration of the game dynamics process.

However, the target loss probability model and Bayesian game model used in project risk assessment models in high-risk areas are based on zero sum non cooperative game theory and incomplete information static game theory, respectively, which have certain limitations. Subsequent research can propose new risk assessment models based on different practical application scenarios and in combination with different game basic theories^26^. The key issue of this model is the identification of a project safety risk index and the screening and quantification of the index. Subsequent research can consider determining risk factors and indicators at various levels through the collection of big data and texts.

## Data Availability

All data generated or analysed during this study are included in this published article.
